# Clinical Usefulness of Right Ventricular–Pulmonary Artery Coupling in Patients with Heart Failure

**DOI:** 10.3390/diagnostics15162083

**Published:** 2025-08-20

**Authors:** Mengyun Yao, Zhenni Wu, Li Zhang, Mengmeng Ji, Shuxuan Qin, Qing He, Yixia Lin, Mingxing Xie, Yuman Li

**Affiliations:** 1 Department of Ultrasound Medicine, Union Hospital, Tongji Medical College, Huazhong University of Science and Technology, Wuhan 430030, China; yaomy@hust.edu.cn (M.Y.); babala0806@163.com (Z.W.); zli429@hust.edu.cn (L.Z.); jimengmeng97@163.com (M.J.); qsxqsx020508@163.com (S.Q.); hqingedu@163.com (Q.H.); linyixia@hust.edu.cn (Y.L.); xiemx@hust.edu.cn (M.X.); 2 Clinical Research Center for Medical Imaging in Hubei Province, Wuhan 430022, China; 3 Hubei Province Key Laboratory of Molecular Imaging, Wuhan 430022, China

**Keywords:** right ventricular-pulmonary artery coupling, heart failure, right ventricular dysfunction, heart failure with preserved ejection fraction

## Abstract

Heart failure (HF) imposes a significant burden on public health, affecting over 56.19 million people worldwide. Right ventricular (RV) dysfunction may occur in HF patients due to various factors, including adverse interventricular interactions, ischemic heart disease, and HF-correlated pulmonary hypertension. Additionally, the deterioration of RV function plays a critical role in the progression of HF, regardless of left ventricular (LV) systolic function, suggesting an unfavorable outcome. Throughout the progression of HF and increasing afterload, the right ventricle undergoes adaptive remodeling to preserve adequate cardiac output. Right ventricular-pulmonary artery (RV-PA) coupling integrates the dynamic adaptation of RV systolic function to afterload and has been considered a stronger predictor of HF prognosis than other conventional parameters. Thus, accurate evaluations of RV-PA coupling are significant in the clinical diagnosis and management of HF patients, along with prognostic speculation. In this review, we summarize the basic principles and measurements of RV-PA coupling and focus on its clinical significance across each subtype of HF.

## 1. Introduction

**Figure 1:**
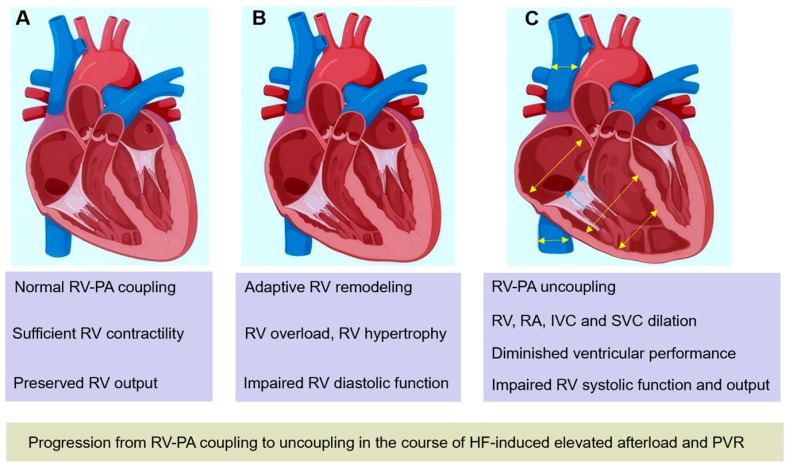
Progression from RV-PA coupling to uncoupling in the course of progressively increasing afterload and PVR caused by HF. (**A**) Normal RV-PA coupling. (**B**) With increasing RV afterload, RV adaptively remodels to maintain sufficient output, RV hypertrophy, diastolic dysfunction, and RV systolic function occur. (**C**) With progressively increasing afterload and PVR, RV dilates to maintain stroke volume, accompanied by tricuspid regurgitation, RA, IVC, and SVC dilation. RV systolic function and output are impaired when RV-PA uncoupling occurs. HF: heart failure; RV, right ventricle; RV-PA, right ventricular-pulmonary artery; IVC: inferior vena cava; PVR: pulmonary vascular resistance; SVC, superior vena cava.

Heart failure (HF) is a life-threatening issue associated with substantial morbidity and mortality, affecting over 56.19 million people worldwide [1]. HF has been categorized into three subtypes based on the left ventricular ejection fraction (LVEF): HF with reduced ejection fraction (HFrEF, LVEF < 40%), mildly reduced ejection fraction (HFmrEF, LVEF between 41% and 49%), or preserved ejection fraction (HFpEF, LVEF ≥ 50%) [2,3]. Right ventricular (RV) function carries substantial prognostic significance in HF, regardless of left ventricular (LV) systolic function [4,5,6]. The right ventricle, being a less muscular chamber, is more sensitive to afterload changes. An elevated left-sided filling pressure caused by LV systolic or diastolic dysfunction is transmitted passively to the pulmonary vessels, resulting in pulmonary hypertension (PH) and subsequent RV dysfunction [7,8]. Right ventricular-pulmonary artery (RV-PA) coupling, defined as the ratio of pulmonary arterial elastance and RV end-systolic function, is a comprehensive parameter that quantitatively indicates the dynamic adaptation of RV systolic function and RV afterload. It has emerged as a stronger predictor of prognosis in HF than other conventional RV function parameters [9,10,11]. In the early stage of PH, the right ventricle undergoes an adaptive response to maintain sufficient cardiac output through contractility enhancement characterized by RV hypertrophy, while RV function remains within the normal range and RV-PA coupling is preserved. However, with progressive PH, right ventricle maladaptive remodeling occurs, featuring chamber dilatation, RV volume increases, RV dysfunction, and even right heart failure [12]. RV-PA uncoupling occurs when the right ventricle fails to generate sufficient contractility to match the RV afterload. Progression from RV-PA coupling to uncoupling in the course of progressively increasing afterload and pulmonary vascular resistance caused by HF is shown in Figure 1. Additionally, RV-PA uncoupling indicates an advanced stage of HF and is a sensitive marker of RV dysfunction [13,14]. Thus, early detection of RV-PA uncoupling plays an important role in both guiding clinical decision-making and predicting prognosis. With the growing interest in RV-PA coupling assessment among HF populations, an increasing number of studies have demonstrated the coupling’s essential impact. In this review, we systematically outline the basic principles and measurements of RV-PA coupling and emphasize its clinical significance in patients with each subtype of HF. 

## 2. Invasive Assessment of Right Ventricular–Pulmonary Artery Coupling

The quantification of RV-PA coupling is complex since determining both load-independent RV contractility and RV afterload simultaneously in one metric is challenging. An invasive approach through right heart catheterization (RHC) can be conducted to describe pressure and volume based on ‘single-beat’ and ‘multi-beat’ pressure–volume loops and is considered the gold standard for assessing RV-PA coupling [15,16,17,18]. The RV contractility and the RV afterload are assessed separately as the end-systolic elastance (Ees) and effective arterial elastance (Ea), and RV-PA coupling is subsequently calculated as the ratio of Ees to Ea as follows:

$$RV-PA\;coupling=\frac{Ees}{Ea}.$$

Ees, representing RV systolic function, is typically calculated as the ratio of end-systolic pressure (ESP) to end-systolic volume (ESV) minus the hypothetical uncompressed ventricular volume (V_0_), as follows:

$$Ees=\frac{ESP}{ESV-V_{0}}.$$

Ea represents a composite of PA resistance, compliance, wave reflection, and RV wall stiffness, which are expressed as the ratio of ESP to stroke volume (SV), as follows:

$$Ea=\frac{ESP}{SV}.$$

**Figure 2:**
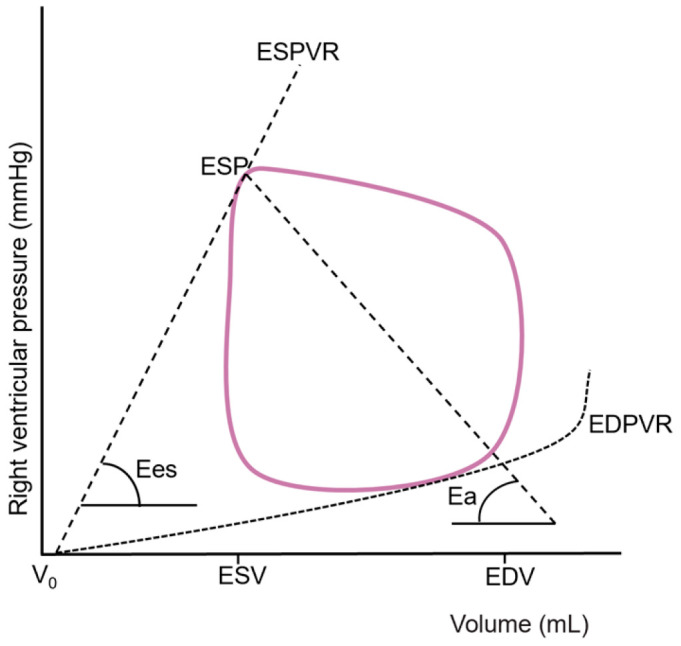
Right ventricular pressure–volume loop from which effective arterial elastance (Ea) and end-systolic elastance (Ees) are derived. EDV: end-diastolic volume; EDPVR: end-diastolic pressure–volume relationship; ESP: end-systolic pressure; ESPVR: end-systolic pressure–volume relationship; ESV: end-systolic volume; V_0_: hypothetical uncompressed ventricular volume.

RV V_0_ is not defined exactly and is generally considered negligible. Thus, RV-PA coupling can also be simplified to the following:

$$RV-PA\;coupling=\frac{SV}{ESV},$$

which turns it into a volumetric index [19,20]. The invasive approach to RV-PA coupling is shown in Figure 2. A coupling ratio of 1.5–2.0 represents the right ventricle maintaining flow output at minimal energy cost [20]. A decrease in Ees/Ea describes relative uncoupling of the RV-PA interaction, indicating the right ventricle failing to adapt to the increased afterload. The Ees/Ea ratio exhibits a certain degree of reserve, indicating that RV volumes may increase until the ratio is 0.8–1.5, but the exact threshold of uncoupling is not defined [18,21,22]. The ratio is preserved in healthy subjects during exercise and at rest but may be impaired in PH or HF populations [23,24,25]. Tello et al. showed that an Ees/Ea cutoff of 0.805 has the ability to detect RV failure in patients with PH [22], while Richter and colleagues reached the conclusion that Ees/Ea < 0.7 is an independent predictor of clinical events [18]. Schmeißer et al. found that Ees/Ea ≥ 0.68 is linked to preserved RV function and mid-term survival in patients with PH due to HFrEF [26]. The invasive ratio of Ees/Ea or SV/ESV is a sensitive and accurate parameter for the detection of the RV adaptation to increasing afterload; however, its clinical application is limited due to its critical technical skill requirements and intrusiveness.

## 3. Non-Invasive Assessment of Right Ventricular–Pulmonary Artery Coupling

Catheter-based measurement of RV-PA coupling has the unavoidable limitations of being invasive, time-consuming, technically demanding, and expensive; therefore, non-invasive alternative metrics have been introduced, in which RV function and afterload are derived non-intrusively. Non-invasive assessments including cardiac magnetic resonance (CMR) and echocardiography are commonly used surrogates of RHC. 

### 3.1. Cardiac Magnetic Resonance Measurement

CMR has emerged as a reliable modality for diagnosing and predicting prognosis in cardiovascular diseases, particularly in HF and PH [18,27,28]. CMR is recognized as the gold standard for assessing RV volumes and EF [29,30,31,32]. As discussed above, Ees/Ea can be simplified to SV/ESV, which can also be acquired by means of CMR and has been confirmed to be strongly correlated with RHC-derived Ees/Ea in patients with pulmonary arterial hypertension [33,34]. In several studies focusing on the prognostic impact of CMR-derived RV-PA coupling, lower SV/ESV values have been demonstrated to be strongly associated with poor outcomes [19,33,34]. However, it should be noted that the simplified formula may underestimate Ees/Ea because of the omission of V_0_.

### 3.2. Echocardiography Measurement

Echocardiography is a well-established tool for qualitatively and quantitatively assessing cardiac structure and function. Owing to its widespread availability, real-time capacity, and clinical convenience, echocardiography has become a frontline imaging modality for assessing RV-PA coupling in cardiovascular diseases. Beyond the commonly used two-dimensional echocardiography (2DE), novel techniques such as three-dimensional echocardiography (3DE) and strain imaging have emerged as reliable tools for evaluating RV-PA coupling.

**Figure 3:**
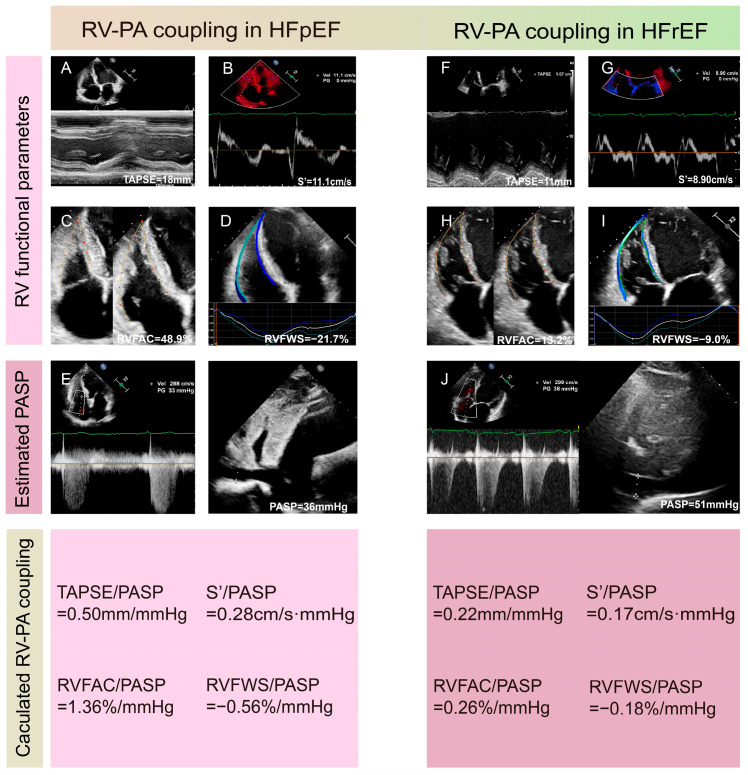
Examples of measurements of right ventricular-pulmonary artery coupling in HFpEF (**A**–**E**) and HFrEF (**F**–**J**) patients using 2DE and speckle tracking echocardiography. (**A**,**F**) Measurements of TAPSE. (**B**,**G**) Measurement of S’. (**C**,**H**) Calculation of RVFAC. (**D**,**I**) Evaluation of RVFWS. (**E**,**J**) Estimation of PASP using peak tricuspid regurgitation velocity, RVSP, and inferior vena cava diameter. 2DE: two-dimensional echocardiography; FAC: functional area change; HFpEF: heart failure with preserved ejection fraction; HFrEF: heart failure with reduced ejection fraction; PASP: pulmonary arterial systolic pressure; RVFWS: free wall longitudinal strain; RVSP: right ventricular systolic pressure; S’: tricuspid annular systolic velocity; TAPSE: tricuspid annular plane systolic excursion.

Tricuspid annular systolic plane excursion (TAPSE) is a common RV systolic function parameter in clinical practice. Pulmonary arterial systolic pressure (PASP) can be acquired through echocardiography and is an estimated index of RV afterload. TAPSE/PASP was first introduced as a surrogate marker for Ees/Ea by Guazzi et al. and has been confirmed to closely correlate with RHC-derived Ees/Ea [35,36]. This indicator has become the most frequently used non-invasive surrogate marker in clinical settings, with multiple studies demonstrating its potent prognostic advantage in tricuspid regurgitation (TR), PH, HF, and congenital heart diseases [18,35,36,37,38,39,40]. The prognostic cutoff value of TAPSE/PASP in HF is about 0.36 mm/mmHg but may differ slightly in different studies and diseases [35,41]. Similarly, other RV systolic function parameters in place of TAPSE are used as non-invasive surrogates, such as the tricuspid annular systolic velocity (S’) and RV functional area change (FAC) measured via 2DE, speckle tracking echocardiography-derived RV free wall longitudinal strain (RVFWS) and RV global longitudinal strain (RVGLS), and 3DE-derived RVEF. All of the above-mentioned indices can be divided by the PASP to serve as non-invasive markers of RV-PA coupling, and they demonstrate predictive value for prognosis in clinical applications [42,43,44,45]. Examples of measurements of RV-PA coupling in HFpEF and HFrEF patients using 2DE and speckle tracking echocardiography are presented in Figure 3. Of note, some recent research suggests that RVFWS/PASP may be a better index, with enhanced diagnostic efficacy, highlighting RVFWS/PASP as a promising indicator for future applications [46].

**Figure 4:**
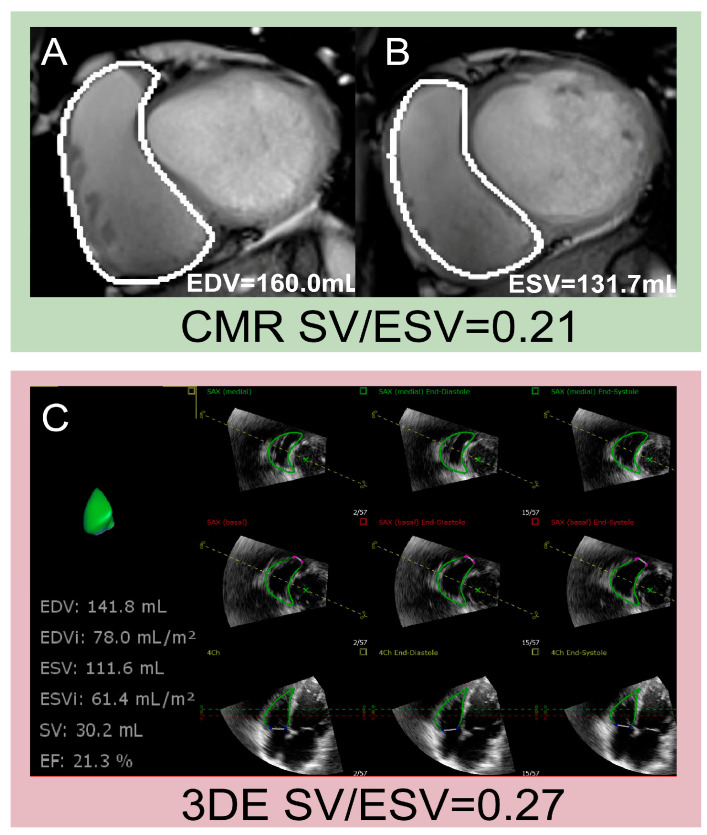
Examples of assessments of SV/ESV using CMR and 3DE. (**A**,**B**) Measurement of EDV, ESV, and SV using CMR; (**C**) measurement of EDV, ESV and SV using 3DE. 3DE: three-dimensional echocardiography; CMR: cardiac magnetic resonance; EDV: end-diastolic volume; ESV: end-systolic volume; SV: stroke volume.

Similar to CMR, 3DE can accurately obtain volume parameters without relying on geometric assumptions of RV morphology. Additionally, 3DE-derived SV/ESV has emerged as a novel parameter of RV-PA coupling, and examples of assessments of SV/ESV using CMR and 3DE are shown in Figure 4. A strong correlation of SV/ESV with Ees/Ea has been confirmed in patients with PH [47]. A few studies have concluded that SV/ESV is a prognostic index in HFpEF and TR [45,48]. Given the limited clinical evidence on 3DE-derived RV-PA coupling in cardiovascular diseases, further studies should explore its diagnostic applicability across various conditions and compare its efficacy with that of other conventional markers. The 2025 ASE guidelines endorse echocardiographic assessments of RV-PA coupling as a valuable adjunct for enhancing risk stratification in clinical practice [49], which emphasizes the need for comprehensive evaluations of RV-PA coupling in the process of evaluating RV function.

## 4. Clinical Usefulness of RV-PA Coupling in Heart Failure

### 4.1. Heart Failure with Preserved Ejection Fraction (HFpEF)

HFpEF is a common subtype of HF, affecting more than half of HF patients, with its prevalence continuing to rise over recent decades [50,51]. It has long been seen as an isolated abnormality in LV diastolic function due to LV hypertrophy and interstitial fibrosis, but numerous studies in recent years have highlighted the fundamental role of RV function and its prognostic value in HFpEF [52,53]. Obokata et al. [54] found that RV structure and function deteriorated more than LV function in patients with HFpEF during a follow-up period of four years, and the development of RV dysfunction was associated with a nearly two-fold increased risk of death. Prior studies confirmed that HFpEF patients are prone to PH and RV dysfunction, with prevalence of nearly 80% and 20–40%, respectively [23,52,53,54,55,56]. The main pathophysiological outcomes related to RV dysfunction and PH include left atrial hypertension caused by backward transmission of high LV filling pressures, pulmonary vascular disease, RV contractile impairment, interventricular interactions, systemic vascular stiffening, and myocardial ischemia [57,58,59]. Therefore, RV-PA coupling, integrating RV contractility and afterload, may serve as a more comprehensive and sensitive indicator for clinical phenotyping, treatment guidance, and prognosis prediction in cases of HFpEF. Studies concerning the application of RV-PA coupling in patients with HFpEF are listed in Table 1.

RV-PA uncoupling represents the terminal stage of HF progression, when a therapeutic approach may be futile. Therefore, identifying these patients in the early stage may be of help in risk stratification and clinical treatment. RV-PA coupling has recently been proposed as a novel parameter to predict outcomes and to help identify different phenotypes. In a study of 528 HFpEF (LVEF ≥ 45%) patients from the PARAGON-HF trial, RVFWS and the RVFWS/PASP ratio were assessed through speckle tracking echocardiography [4]. After a median follow-up of 2.8 years, the researchers concluded that lower absolute RVFWS and RVFWS/PASP ratio values were correlated with higher NT-pro brain natriuretic peptide (BNP) levels and higher prevalence of adverse outcomes of HF hospitalizations and cardiovascular death [4]. Likewise, Nakagawa et al. [67] conducted a multi-center Asian cohort study of 655 individuals, enrolling acute decompensated HFpEF patients and demonstrating that TAPSE/PASP < 0.48 mm/mmHg was independently associated with HF readmission and all-cause mortality. Their study further identified that RV-PA uncoupling in HFpEF patients was correlated with renal dysfunction, higher NT-pro BNP levels, impaired exercise tolerance, and shortened 6 min walk distances [67]. Similarly, emerging evidence underscores the critical role of RV-PA coupling in HFpEF phenotyping to guide therapeutic strategies [74,75]. Guazzi and colleagues analyzed 387 HFpEF patients (of which 219 individuals underwent RHC) [63] to validate TAPSE/PASP against invasive RV-PA coupling measures (Ees/Ea). They observed that, from the highest to the lowest tertiles of the TAPSE/PASP ratio, there was a gradual increase in the levels of BNP, deterioration of systemic and pulmonary hemodynamics, abnormal exercise oxygen consumption, and reduced ventilation efficiency [63]. Moreover, the lowest tertile of TAPSE/PASP had lower PA compliance, higher pulmonary vascular resistance, and a higher incidence of clinical worsening, which supports the assertion that the impact of PH on adverse outcomes is related to RV-PA coupling, regardless of LV systolic function [63]. The strong correlation between TAPSE/PASP and Ees/Ea supports its utility as a reliable non-invasive marker of RV-PA coupling. This study emphasizes the possible application of the non-invasive index TAPSE/PASP as a convenient method for stratifying HFpEF phenotypes and predicting prognosis. Considering that HFpEF patients may experience normal hemodynamics but suffer multiple cardiac abnormalities during exercise [52], another recent study by Saito and colleagues aimed to identify HFpEF phenogroups through exercise echocardiography-based machine learning cluster analysis [76]. Two hundred and sixty-five enrolled HFpEF patients were categorized into three phenogroups: phenogroup 1, with preserved biventricular systolic reserve, the highest cardiac output, and RV-PA coupling during exercise; phenogroup 2, with a higher prevalence of atrial fibrillation, increased PA and RV pressure, impaired RV functional reserve, the most severe left atrial remodeling, RV-PA uncoupling, and RV failure during exercise; and phenogroup 3 with ventricular and arterial stiffness, impaired LV diastolic reserve, and impaired exercise capacity [76]. Phenogroups 2 and 3 had a three-fold increased risk of composite outcomes when compared with phenogroup 1. The study further demonstrated that the cluster analysis based on exercise echocardiography had incremental prognostic value over the conventional 2DE parameters and may be a promising tool not only in diagnosis but as a risk stratification tool in HFpEF. The studies by Guazzi and Saito et al. both indicated a higher prevalence of atrial fibrillation in HFpEF patients with the lowest TAPSE/PASP values [63,76]. Gorter et al. compared RV function and RV-PA coupling in HFpEF patients with sinus rhythm and atrial fibrillation through 2DE and RHC [65]. Their conclusion that RV-PA coupling was lower in patients with HFpEF combined with atrial fibrillation was consistent with the findings of the studies above [65].

Since PH is common in HFpEF patients and is a strong predictor of poor outcomes, early detection and differentiation of PH may be beneficial to enhancing the prognosis of HFpEF. Chen and colleagues conducted a trial on HFpEF using the calculated TAPSE/PASP and S’/PASP values both during exercise and at rest [70]. Lower resting/exercise RV-PA coupling was found in HFpEF patients with PH compared with those without PH. Both TAPSE/PASP and S’/PASP were associated with mPAP and pulmonary capillary wedge pressure. The researchers concluded that the cutoff points to differentiate HFpEF patients with PH from those without PH l were TAPSE/PASP ≤ 0.62 mm/mmHg and S’/PASP ≤ 0.47 cm/s per mmHg [70]. Thus, a decline in TAPSE/PASP and S’/PASP in the context of HFpEF may be suggestive of appropriate diagnostic procedures to guide clinical treatment.

Other than helping to stratify phenotypes and predict outcomes in HFpEF patients, RV-PA coupling is a powerful tool to guide clinical management. A prospective trial conducted by Andersen et al. enrolled 39 HFpEF patients and 18 controls, all of whom underwent comprehensive hemodynamic assessments by means of both RHC and echocardiography at rest and during dobutamine infusion [60]. Compared with the control group, dobutamine infusion in HFpEF patients led to a more pronounced reduction in pulmonary arterial resistance, increased pulmonary arterial compliance, and a more negative slope in the PA pressure–flow relationship, which indicated that the pulmonary vascular damage in the early stage was reversible [60]. The dynamic RV-PA coupling represented by S’/mean PA pressure (mPAP) revealed that the improvement in RV ejection during dobutamine infusion in HFpEF was caused by afterload relief rather than contractility enhancement. The study then summarized that the favorable pulmonary vascular response to dobutamine in early-stage HFpEF may improve RV-PA coupling and provide evidence for β-stimulation as a potential treatment approach for HFpEF [60]. Another study by Reddy et al. enrolled 37 randomized participants to evaluate the effect of dapagliflozin on RV-PA coupling and pulmonary vascular load at rest and during exercise [71]. Dapagliflozin was able to improve the prognosis and health condition by decreasing the abnormal pulmonary capillary wedge pressure of HFpEF patients during exercise [77]. Former studies saw the role of dapagliflozin in ameliorating metabolic dysfunction and inflammation, but the exact mechanisms remained unknown. The researchers then hypothesized that dapagliflozin could improve the pulmonary vascular load and RV-PA coupling during exercise to achieve this therapeutic goal. The conclusion made after 24 weeks of dapagliflozin treatment were consistent with this hypothesis. During exercise, the pulmonary vascular load was reduced, while pulmonary arterial compliance and RV-PA coupling were enhanced; these enhancements were correlated with reductions in right atrial and pulmonary capillary wedge pressure, together with improvement in load-independent RV functional reserve [71]. The improvement in RV-PA coupling may partially explain the benefits observed with dapagliflozin in HFpEF treatment beyond the left heart. Therefore, monitoring dynamic changes in RV-PA coupling during clinical treatment could predict therapeutic response, since it may be useful in identifying a patient cohort that is sensitive to a certain treatment.

### 4.2. Heart Failure with Mildly Reduced Ejection Fraction (HFmrEF)

HFmrEF is a type of HF that has gradually attracted more attention in recent years as its prevalence has been increasing to 10–25% among the overall population of patients with HF [78]. The pathophysiological mechanisms in HFmrEF patients are relatively complex and may include mild impairment of LV systolic function, diastolic dysfunction, or abnormalities in right heart function and pulmonary circulation [79,80]. Patients with HFmrEF may have a similar or higher risk of non-cardiovascular adverse events to patients with HFrEF and higher rates of cardiovascular mortality than those with HFpEF [79]. This indicates that HFmrEF patients have an elevated risk of disease progression and require more aggressive management and treatment [79]. 

RV-PA coupling indices can serve as independent predictors for the prognostic assessment of HFmrEF patients, facilitating early identification of high-risk patients and enabling timely therapeutic strategy optimization, ultimately improving clinical outcomes. Multiple studies have shown that RV-PA coupling indices are closely related to the prognosis of HFmrEF patients [6,79,80,81]. A retrospective investigation that enrolled 400 outpatients with chronic HF aged over 70 years and a reduced mid-range ejection fraction (LVEF ≤ 50%) showed that TAPSE/PASP outperformed LV function parameters in predicting the prognosis of these HF patients, with an optimal threshold of 0.34 mm/mmHg [81]. A study conducted by Ghio et al. [6] involving 1663 HF patients (1123 with HFrEF, 156 with HFmrEF, and 384 with HFpEF) reported that the TAPSE/PASP ratio was an independent predictor of prognosis for all types of HF patients, regardless of the degree of left ventricular systolic dysfunction.

Although a growing number of studies have demonstrated the prognostic value of RV-PA coupling in patients with HFmrEF, research on the usefulness of RV-PA coupling in HFmrEF is still in the preliminary stage. Further in-depth studies on its mechanisms and clinical application are needed in the future to better guide clinical decision-making.

### 4.3. Heart Failure with Reduced Ejection Fraction (HFrEF)

HFrEF is a common type of HF, with complex pathophysiological mechanisms involving multiple structural and functional abnormalities. RV-PA coupling, as an important physiological mechanism of cardiopulmonary circulation, plays a significant role in the progression and prognosis of HFrEF. Studies concerning the application of RV-PA coupling in patients with HFrEF are listed in Table 2.

RV-PA coupling is of great significance in assessing the clinical progression and prognosis of HFrEF. Preserved RV-PA coupling is essential for maintaining stable cardiac function, while RV-PA uncoupling indicates pathological remodeling, disease progression, and adverse clinical outcomes. A study involving 97 patients with HFrEF showed that although the patients in group B (those with TAPSE at rest of < 16 mm and median TAPSE ≥ 15.5 mm at peak exercise) and group C (those with TAPSE at rest of < 16 mm and median TAPSE < 15.5 mm at peak exercise) had similar LVEFs and resting RV impairment, those in group B showed some degree of right ventricular exercise contractile reserve (RVECR) (upward shift of the length–force relationship), better RV-PA coupling (lower mean pulmonary artery pressure vs. cardiac output slope), and greater ventilator efficiency (lower slope of minute ventilation vs. carbon dioxide output) [83]. Thus, an unfavorable RV contractile adaptive response to exercise might not always be caused by impaired RV function at rest. Evaluating the degree of RVECR and RV-PA coupling during exercise could be useful and unmask various clinical phenotypes and different levels of risk even in the more advanced stages of HF. Legris et al. [84]. demonstrated that RV function rather than LV function was closely associated with exercise capacity in ambulatory HFrEF patients, providing new evidence of the importance of RV function and RV-PA coupling in exercise tolerance in HFrEF. The TAPSE/PASP ratio was the only echocardiographic parameter associated with peak VO_2_; specifically, a ratio threshold of ≤0.45 mm/mmHg predicted peak VO_2_ ≤ 14 mL/kg/min and a threshold of ≤ 0.39 mm/mmHg predicted peak VO_2_ ≤ 12 mL/kg/min. This study indicated that TAPSE/PASP could be used to identify patients with reduced exercise capacity.

Multiple studies have shown that impaired RV-PA coupling is closely related to poor prognosis in patients with HFrEF [92,93]. In a 2.39-year follow-up study of 456 patients with ventricular–secondary mitral regurgitation caused by HFrEF, RV-PA coupling calculated as the TAPSE/PASP ratio was used to evaluate the compensatory increase in pulmonary pressure. The study showed that RV-PA uncoupling (TAPSE/PASP ratio < 0.37 mm/mmHg) was associated with reduced survival across all severities of mitral regurgitation [85]. Similarly, Hădăreanu et al. found that RVFWS/PASP is a robust prognostic marker in HFrEF–secondary mitral regurgitation, with a cutoff of  0.46%/mmHg [94]. An investigation that involved 4312 patients with HF showed that the RVGLS/PASP ratio was a significant predictor of all-cause mortality, including HFrEF (HR, 2.124; *p* = 0.002), HFmrEF (HR, 2.733; *p* = 0.021), and HFpEF (HR, 2.134; *p* = 0.006), and an RVGLS/PASP ratio of ≤0.32%/mmHg was associated with an increased risk of mortality among all the HF phenotypes [87]. A study that involved 315 patients with an LVEF of <45% showed that RVGLS/PASP and RVFWS/PASP were significantly and independently associated with increased mortality risk. Moreover, the accuracy of the combined assessment of RVGLS/PASP and RVFWS/PASP was greater than that of the parameters evaluated separately, which could be used to identify high-risk patients. The prognostic accuracy of RVGLS/PASP and RVFWS/PASP was higher than that of TAPSE/PASP [89].

Although TAPSE/PASP is a convenient index to evaluate RV-PA coupling, it also has limitations that cannot be ignored. Under circumstances of inadequate visualization of the inferior vena cava and/or the tricuspid regurgitation velocity (TRV) Doppler signal, TAPSE/PASP may not be feasible. A cohort study of 200 hospitalized HF patients used alternative measures of RV-PA coupling parameters, namely the product of TAPSE and pulmonary acceleration time (TAPSE × pACT) and the ratio of TAPSE to the peak tricuspid regurgitation velocity (TAPSE/TRV), for prognostic assessment in HF [86]. They demonstrated that TAPSE/PASP was the most powerful predictor of mortality, followed by TAPSE × pACT and TAPSE/TRV, and both had strong correlations with the TAPSE/PASP ratio [86].

As TAPSE exclusively quantifies longitudinal contraction, the TAPSE/PASP ratio might serve as a suboptimal surrogate for RV-PA coupling in patients with advanced RV structural remodeling. A prospective study enrolling 105 outpatients with dilated cardiomyopathy (DCM) and a mean LVEF of 28 ± 7% showed that the RV SV/ESV ratio was independently correlated with severe HF symptoms in patients with DCM, and the cutoff value was 0.54 (area under the curve = 0.712, *p* < 0.001) for predicting severely symptomatic status; thus, it might be a useful risk stratification tool for these patients [88]. Aura et al. assessed RV-PA coupling in 60 patients with DCM and LVEF < 40% using five echocardiographic parameters: TAPSE/PASP, RVGLS/PASP, RVFWS/PASP, 3D RVEF/PASP, and RV SV/ESV. They found that RVFWS/PASP and RVEF/PASP were independent predictors of HF rehospitalization in patients with DCM. Cutoff values of RVFWS/PASP > −0.40%/mmHg and RVEF/PASP < 1.30%/mmHg were proposed to predict a high risk of HF rehospitalization [90]. Therefore, early identification and intervention in cases of abnormal RV-PA coupling have potential clinical value in improving the prognosis of patients with HFrEF.

In addition, RV-PA coupling can be used to guide treatment decisions. Currently, the treatment strategies in patients with HFrEF mainly include pharmacological therapy and device-based therapy. In terms of pharmacological therapy, sacubitril/valsartan is a novel angiotensin receptor neprilysin inhibitor (ARNI), which can improve the contractile function of the right ventricle, ameliorate RV relaxation, and reduce pulmonary artery pressure, thereby partially restoring RV-PA coupling. Daniele Masarone et al. found that sacubitril/valsartan could improve RV-PA coupling in patients with HFrEF, as evidenced by an increase in TAPSE and a decrease in PASP. The improvement in RV-PA coupling was related not to LV reverse remodeling but only to reduction in the left atrial volume index [82]. This suggests that when treating HFrEF, it is possible to consider using this type of drug to optimize the treatment plan based on the patient’s RV-PA coupling status to improve RV function and alleviate PH. Regarding device-based therapy, such as cardiac resynchronization therapy (CRT), this method can optimize the electrical activity and mechanical contraction of the heart, which may have a positive impact on RV-PA coupling, but more than 1/3 of patients do not respond to CRT [95]. Therefore, there is a strong need to identify new factors to predict and/or influence the response of HFrEF to CRT. In a prospective investigation including 54 patients with HFrEF undergoing CRT, a TAPSE/PASP ratio of ≥ 0.58 mm/mmHg displayed good sensitivity (90%) and specificity (81.8%) for predicting CRT response. Moreover, a TAPSE/PASP ratio of < 0.58 mm/mmHg was associated with a higher incidence of death and HF hospitalizations during the follow-up period [91]. Hence, by monitoring changes in RV-PA coupling after treatment, the therapeutic effect can be evaluated, and treatment strategies can be adjusted accordingly. 

## 5. Summary and Prospects

The application of RV-PA coupling in different types of HF holds significant clinical importance. RV-PA coupling can be used to assess exercise tolerance, guide treatments, monitor treatment efficacy, and stratify risk in HF patients.

Despite the advancements made in understanding RV-PA coupling within the context of HF, several challenges and future research directions remain. First, the current methods for assessing RV-PA coupling are diverse, including both invasive and non-invasive approaches, each with its own advantages and disadvantages. Future efforts should focus on further optimizing these assessment methods to improve their accuracy and reproducibility, thereby facilitating better application in clinical practice. Second, the research on RV-PA coupling in HFmrEF is relatively limited at present. More studies are needed to better understand its role and value in this type of HF. In addition, the potential applications of RV-PA coupling in HF treatment warrant further exploration. For instance, interventions targeting RV-PA coupling may offer new therapeutic strategies for HF patients. Future research could investigate how to improve RV-PA coupling through pharmacological therapy, device-based therapy, or other interventions, thereby improving the prognosis of HF patients. Lastly, long-term follow-up studies examining changes in RV-PA coupling among HF patients hold considerable importance. By monitoring these changes over time, we can better understand their relationship with the progression and prognosis of HF, which will help in formulating more personalized treatment plans and improving the long-term survival and quality of life of HF patients.

## Figures and Tables

**Table 1:** Studies concerning the application of right ventricular-pulmonary artery coupling in HFpEF patients.

Reference	Publication Year	Sample Size	RV-PA Coupling Indices	Median Follow-Up	Main Results
Melenovsky et al. [53]	2014	142	RV FAC/mPAP	529 days	RV function was impaired in HFpEF using both load-dependent (RV FAC) and load-independent parameters (RV FAC/mPAP).
Andersen et al. [60]	2015	57	S’/mPAP	-	Dobutamine improved RV ejection in HFpEF subjects through afterload reduction rather than increasing contractility confirmed by dynamic analysis of RV-PA coupling.
Borlaug et al. [61]	2016	74	-	-	During exercise, RV reserve was impaired in HFpEF patients in association with high filling pressures and inadequate cardiac output responses. The relationship between PA pressure and flow was steeper, which indicated the RV afterload increased for any given cardiac output.
Hussain et al. [62]	2016	137	TAPSE/PASP	24 weeks	HFpEF with RV dysfunction and RV-PA uncoupling tended to experience more advanced HF, for whom treatment with sildenafil for 24 weeks did not increase RV-PA coupling, exercise capacity, or ventilatory efficiency.
Guazzi et al. [63]	2017	387	TAPSE/PASP	13.4 months	Tertile 1 with a TAPSE/PASP ratio < 0.35 mm/mmHg had a higher prevalence of atrial fibrillation and kidney dysfunction and experienced lower PA compliance and higher pulmonary vascular resistances. TAPSE/PASP was confirmed as an independent prognosis index and a novel parameter to help stratify HFpEF phenotypes at different level of risk.
Bosch et al. [64]	2017	657	RVFWS/PASP, TAPSE/PASP	715 days	Both RVFWS/PASP and TAPSE/PASP were correlated with all-cause mortality and HF hospitalization in HF patients regardless of LVEF. The cutoff values were TAPSE/PASP < 0.48 mm/mmHg and RVFWS/PASP ≥ −0.56%/mmHg.
Gorter et al. [65]	2017	91	TAPSE/PASP	-	A lower index of TAPSE/PASP was found in HFpEF patients with current atrial fibrillation. Atrial fibrillation was associated with RV dysfunction independently from pulmonary pressures.
Gorter et al. [39]	2018	97	TAPSE/PASP	816 days	TAPSE/PASP < 0.36 mm/mmHg could identify HFpEF patients with additional pre-capillary PH. Worsening of TAPSE/PASP was a predictive factor of poor outcome.
Singh et al. [23]	2019	88	Ees/Ea	-	RV-PA uncoupling caused by both RV contractility impairment and afterload mismatch was common in HFpEF patients. Resting and dynamic RV-PA uncoupling in HFpEF was driven by an increase in RV pulsatile rather than resistive afterload. However, RV-PA uncoupling deteriorated dynamically during exercise with the addiction effects of increased RV resistive afterload.
Santas et al. [66]	2019	760	TAPSE/PASP	2 years	TAPSE/PASP < 0.36 mm/mmHg was associated with a higher risk of HF rehospitalizations, patients with lowest index (TAPSE/PASP < 0.28 mm/mmHg) underwent highest risk of all-cause and HF-related recurrent admissions. RV-PA coupling was a strong predictor of rehospitalizations of HFpEF.
Nakagawa et al. [67]	2020	655	TAPSE/PASP	333 days	RV-PA uncoupling was associated with the composite endpoint and all-cause mortality. TAPSE/PASP < 0.48 mm/mmHg was significantly associated with clinical outcomes of acute decompensated patients with HFpEF and may suffer higher prevalence of renal dysfunction and worse exercise capacity.
Singh et al. [24]	2021	75	Ees/Ea	-	Male patients tended to have more impaired RV-PA coupling with reduced peak VO2 compared with female patients driven by RV contractility impairment and afterload mismatch. On the contrary, female HFpEF patients showed better performance in peak exercise VO2 and preserved RV-PA coupling than male patients.
Pugliese et al. [68]	2022	220	TAPSE/PASP	25 months	Pronounced impairment in RV-PA coupling was found in HFpEF patients than HFrEF patients during exercise. HFpEF patients displayed a higher prevalence of systemic inflammation.
Inciardi et al. [4]	2023	528	RVFWS/PASP	2.8 years	Decreased absolute RVFWS and RVFWS/PASP ratios were both related to elevated NT-pro BNP and were found to be significantly associated with negative outcomes of HF hospitalizations and cardiovascular death in HFpEF patients.
Jia et al. [69]	2023	250	TAPSE/PASP	433 days	TAPSE/PASP ≤ 0.43 mm/mmHg was an independent prognostic factor of adverse outcomes for the primary endpoint, all-cause death, and recurrent HF hospitalization in acute HFpEF patients with coronary artery disease.
Chen et al. [70]	2024	113	TAPSE/PASP S’/PASP	-	Patients with HFpEF with PH had lower RV-PA coupling than those without PH both at rest and during exercise. Decreased TAPSE/PASP and S’/PASP were related to with abnormal rest/exercise pulmonary hemodynamics in patients with HFpEF, and the two indexes could thus identify HFpEF patients with PH with cutoff values of ≤0.62 and ≤0.47, respectively.
Reddy et al. [71]	2024	37	S’/mPAP	24 weeks	Dapagliflozin treatment for 24 weeks could favorably improve RV-PA coupling and reduce PA loading during exercise among HFpEF patients, probably benefiting from the reduction pulmonary capillary wedge pressure.
Lechuga et al. [72]	2025	15	Ees/Ea	-	In HFpEF patients with PH, a negative relation was found between characteristic pulmonary impedance and Ees/Ea index during exercise, which was not evident in precapillary PH.
Decotto et al. [73]	2025	142	TAPSE/PASP	1 year	In elderly patients aged ≥ 75 years with HFpEF, RV-PA uncoupling was in association with adverse outcome during 1-year follow-up.

Ees: end-systolic elastance; Ea: effective arterial elastance; FAC: functional change area; HF: heart failure; HFpEF: heart failure with preserved ejection fraction; HFrEF, heart failure with reduced ejection fraction; LVEF: left ventricular ejection fraction; mPAP: mean pulmonary artery pressure; Nt-pro BNP: Nt-pro brain natriuretic peptide; PA: pulmonary artery; PASP: pulmonary artery systolic pressure; PH: pulmonary hypertension; RV-PA coupling: right ventricular-pulmonary artery coupling; RV: right ventricular; RVFWS, right ventricular free wall longitudinal strain; S’: tricuspid annular systolic velocity; TAPSE: tricuspid annular systolic plane excursion.

**Table 2:** Studies concerning the application of right ventricular-pulmonary artery coupling in HFrEF patients.

Reference	Publication Year	Sample Size	RV-PA Coupling Indices	RV-PA Uncoupling Cutoff	Median Follow-Up	Main Results
Masarone et al. [82]	2020	163	TAPSE/PASP	-	2 years	Sacubitril/valsartan could improve the RV-PA coupling in patients with HFrEF.
Guazzi et al. [83]	2016	97	TAPSE/PASP mPAP/CO slope	-	16 months	Testing the degree of RVECR and RV-PA coupling during exercise could be useful and unmask various clinical phenotypes and different levels of risk even in the more advanced stages of HF.
Legris et al. [84]	2022	205	TAPSE/PASP	TAPSE/PASP ≤ 0.45 mm/mmHg	-	RV function was closely associated with exercise capacity in ambulatory HFrEF patients, providing new evidence on the importance of RV function and RV-PA coupling (TAPSE/PASP) on exercise tolerance in HFrEF.
Watson et al. [85]	2024	456	TAPSE/PASP	TAPSE/PASP < 0.37 mm/mmHg	2.39 years	RV-PA uncoupling (the median TAPSE/PASP ratio < 0.37 mm/mmHg) was associated with reduced survival across all severities of mitral regurgitation.
Pestelli et al. [86]	2021	200	TAPSE × pACT TAPSE/TRV	TAPSE ×pACT < 140 cm·ms TAPSE/TRV < 5.5ms	2.7 years	TAPSE/PASP was the most powerful predictor of mortality, followed by TAPSE × pACT and TAPSE/TRV, and the both have high correlations with TAPSE/PASP ratio.
Park et al. [87]	2022	4312	RVGLS/PASP	RVGLS/PASP ≤ 0.32 %/mmHg	35.0 months	The RVGLS/PASP ratio ≤ 0.32 %/mmHg was associated with an increased risk of mortality in all the HF phenotypes.
Vîjîiac et al. [88]	2022	139	SV/ESV	SV/ESV < 0.54	-	3D SV/ESV was an independent correlate of severe HF symptoms in patients with DCM, and the cutoff value was 0.54 for predicting severely symptomatic status, which might be a useful risk stratification tool for these patients.
Iacoviello et al. [89]	2017	315	RVGLS/PASP, RVFWS/PASP and TAPSE/PASP	RVGLS/PASP < 0.36 %/mmHg RVFWS/PASP < 0.65 %/mmHg	36 ± 26 months	RVGLS/PASP and RVFWS/PASP were significantly and independently associated with an increased mortality risk.
Vîjîiac et al. [90]	2022	60	TAPSE/PASP; RVGLS/PASP; RVFWS/PASP; 3D RVEF/PASP and 3D RV SV/ESV.	RVFWS/PASP > −0.40 %/mmHg RVEF/PASP < 1.30 %/mmHg	18 months	RVFWS/PASP and RVEF/PASP were independent predictors of HF rehospitalization in patients with DCM.
Ghio et al. [6]	2017	1663	TAPSE/PASP	TAPSE/PASP < 0.36 mm/mmHg	-	The TAPSE/PASP ratio was an independent predictor of prognosis for all types of HF patients, regardless of the degree of left ventricular systolic dysfunction.
Deaconu et al. [91]	2021	54	TAPSE/PASP	TAPSE/PASP < 0.58 mm/mmHg	31 ± 12.9 months	The ratio TAPSE/PASP could predict the CRT response. The ratio TAPSE/PASP < 0.58 mm/mmHg was associated with a higher incidence of death and HF hospitalizations.

HF, heart failure; HFrEF, heart failure with reduced ejection fraction; PASP, pulmonary artery systolic pressure; RV, right ventricle; CRT, cardiac resynchronization therapy; RVECR: right ventricular exercise contractile reserve; RVGLS, right ventricular global longitudinal strain; RVFWS, right ventricular free wall longitudinal strain; TAPSE, tricuspid annular plane systolic excursion; TTE, transthoracic echocardiography; CO, cardiac output; mPAP, mean pulmonary artery pressure; SV, stroke volume; ESV, end-systolic volume; DCM, dilated cardiomyopathy; pACT, pulmonary acceleration time; TRV, peak tricuspid regurgitation velocity; RVEF, right ventricular ejection fraction; 3D, three-dimensional.

## Data Availability

No data were generated or analyzed for or in support of this paper. We did not use artificial intelligence (AI)-assisted technologies in the production of the submitted work.

## Abbreviations

The following abbreviations are used in this manuscript:

ARNI	angiotensin receptor neprilysin inhibitor
Ea	effective arterial elastance
Ees	end-systolic elastance
HFmrEF	heart failure with mildly reduced ejection fraction
HFpEF	heart failure with preserved ejection fraction
HFrEF	heart failure with reduced ejection fraction
pACT	pulmonary acceleration time
PASP	pulmonary arterial systolic pressure
PH	pulmonary hypertension
RHC	right heart catheterization
RVECR	right ventricular exercise contractile reserve
RV-PA coupling	right ventricular-pulmonary artery coupling
RVFWS	right ventricular free wall longitudinal strain
RVGLS	right ventricular global longitudinal strain
TRV	tricuspid regurgitation velocity
